# Understanding the Golgi Apparatus and Intracellular Transport Pathways

**DOI:** 10.3390/ijms24087549

**Published:** 2023-04-20

**Authors:** Alexander A. Mironov

**Affiliations:** Department of Cell Biology, IFOM ETS—The AIRC Institute of Molecular Oncology, Via Adamello, 16, 20139 Milan, Italy; alexandre.mironov@ifom.eu

Today, the future paradigm of intracellular transport could be based on four competing models, namely the vesicular model, the cisterna maturation–progression model, the diffusion model, and the kiss-and-run model. In the current Special Issue of the *International Journal of Molecular Science* (*IJMS)*, seven papers were published. In this editorial, I aim to summarize important discoveries in the field of intracellular transport which have considerably contributed to the understanding of the function and organization of the Golgi complex (GC) and introduce new findings presented in the current Special Issue of the *International Journal of Molecular Science*.

In this Special Issue, two original papers are presented by Beznoussenko et al. [1,2]. In these papers, models of intra-Golgi transport are compared. Three papers are presented by Mironov et al. [3,4,5]. These papers represent reviews devoted to the role of intracellular transport and models describing this process for different tissue cells and the role of intracellular transport for COVID-19. The review paper by Brodsky et al. [6] explores the role of the GC in the organization of microtubules. Finally, the paper by Dejgaard and Presley [7] discusses the roles of membrane trafficking in the life cycle of lipid droplets. This includes the complementary roles of lipid phase separation and proteins in the biogenesis of lipid droplets from endoplasmic reticulum (ER) membranes, and the attachment of mature lipid droplets to membranes by lipidic bridges and by more conventional protein tethers.

The essence of different models of intra-Golgi transport and their problems are described in detail by Mironov and Beznoussenko [8]. The progression model (or the concept of cis-to-trans flow) was the first mechanism proposed for the explanation of mechanisms of intracellular transport. The diffusion mechanism could be based not only on the constantly existing wide connections, but also on narrow connections in combination with the bolus-like mechanism [9]. The main problems of the lateral diffusion model are the presence of SNARE complexes within all steps of the secretory and endocytic pathway and the existence of gradients across the Golgi. The bolus-like mechanism was adapted for intra-Golgi transport in the form of peristaltic movement of membranes [10].

However, if the connections are transient, one can use with the kiss-and-run model (KARM) that does not face problems due to the existence of SNAREs. The KARM assumes that compartments fuse with each other, and then become separated from each other. KARM has been proposed for synaptic vesicles, for the fusion of secretory granules with the plasma membrane in neuroendocrine cells, and fusion between endosomes and lysosomes [11].

For its normal function, the KARM has several requirements.

1. To ensure that correct compartments will fuse with each other, it is necessary to have a mechanism for SNARE sorting along the secretory and endocytic pathways.
2. There should be a working mechanism for the concentration of SNARE in sites through which two compartments fuse with each other.
3. The cells have to have a mechanism to break connections.
4. The connections between organelles should be thin. If connections become thick, it will be necessary to perform their fission initially to make them thin. On the other hand, if connections become thick, the ionic composition of two compartments involved in kiss-and-run mechanism will be easily equilibrated.
5. Cells should have a mechanism to stimulate fusion at the defined time.
6. It should be a gradient of ionic pumps of other protein machineries regulating the concentration of ions along the secretory and endocytic pathway able to create linear gradients.
7. The kiss-and-run mechanism actually means that there is no specific retrograde transport, and this transport occurs simultaneously with the anterograde transport.

In the framework of the kiss-and-run model, the three main coats have different functions. The important role of coat-dependent concentration through the cytosolic domain of membrane proteins is the concentration of SNAREs at the correct locations of endomembrane compartments.

On the other hand, the kiss-and-run mechanism can exist in two forms: the first is when two organelles temporarily fuse with each other and then become separated, and the second mechanism involves the temporary fusion between two organelles in one site with consecutive fission in another places, thus causing some membrane displacement from one organelle to another. The carrier maturation model is the individual case (the asymmetric KARM) within the frame of the kiss-and-run mechanism.

In the framework of the KARM, the fission of membrane tubules connecting different Golgi compartments becomes one of the most important mechanisms. There are two possibilities here—the first is that the machineries responsible for fission do not exist, and the second is that only local temperature fluctuations can regulate fission.

The main challenge for the KARM is the elucidation of the function of coat complexes. The most enigmatic issue is the role of COPI vesicles. First of all, COPI vesicles are not an obligatory feature of all cells. In some eukaryotes, intracellular transport can occur without the generation of COPI-dependent vesicles. For instance, in minimal secretory (Microsporidia) systems, 50–60 nm vesicles (both COPI- and COPII-dependent) do not exist at all because both COPI and COPII machineries in this parasite are reduced [12].

Several roles have been proposed for COPI-dependent vesicles. COPI vesicles can impact the regulation of the cisterna shape [13] or the generation of COPI-coated buds in order to facilitate the subsequent uncoating of Golgi membranes [8]. Transformation of COPI-coated buds into vesicles could accelerate the uncoating of Golgi membranes from COPI. COPI participates in the fission of COPI-coated Golgi buds [14]. The same mechanism may be responsible for the fission of intercisternal connections. COPI is involved in the formation of cisternal pores [1,2,8].

In 2007, S. Rothman [15], who was against the vesicular model from the very beginning [16], wrote: “…it (protein synthesized in the ER) must exit by the budding of membrane because no other option exists”. However, now, such an option exists—the KARM [8,11]. Today, the most powerful model for the explanation of the ER-to PM transport is the KARM. However, in order to confirm its role as a paradigm, a high volume of experiments and re-interpretations should be performed. The KARM can be symmetric, asymmetric for membrane cargoes and even spiral-based. The KARM explains why the secretory and endocytosis pathways appear as a united system. However, there are many unresolved questions necessary for the explanation of intracellular transport, especially of regulation secretory proteins. Its main pitfalls include the function of COPI vesicles, the mechanisms of SNARE recycling and the rare presence of intercisternal connections within Golgi stacks.
